# A Lady Cook

**Published:** 1887-10-29

**Authors:** 


### The Matrons' Corner.

[Replies, endorsed "Matron," should be addressed to the Editor.]

### A LADY COOK.

A Resting Matron could not give the name of any special hospital where a lady could try the experiment of being cook, but if she would state her qualifications and willingness in The Hospital, some married matrons would be thankful for her services. As to the duties, she would have to encounter similar difficulties to those of Miss Nightingale in the nursing. Miss Nightingale objected to nothing, however disagreeable, but by her tact and intelligence showed that it would be wasting her time to be cleaning grates, etc. Take a lady cook now into any hospital : no lifting of weights is (to my knowledge) ever expected. A porter is always kept who can hang up the meat and lift the joints in and out of the oven by a little management. The same thing about the milk—the cook simply measures it. A kitchenmaid is always kept, who does the cleaning and washing up ; so, except washing the bowls, tables, etc., which the cook uses herself, that difficulty is overcome. Next the meals. A wise woman would always preside at the servants' dinner-table. Her tea and supper she could arrange for herself. If she had travelled abroad, and seen how simply people live, she could invent some comfortable method. The early rising is only what the nurses share. Her position would be respected from the first, but in time her friends would be innumerable. X.

